# The Detection of Physiological Changes Using a Triaxial Accelerometer and Temperature Sensor-Equipped Bolus-Type Biosensor in Calves

**DOI:** 10.3390/ani14192815

**Published:** 2024-09-29

**Authors:** Leegon Hong, Younghye Ro, Atsushi Kimura, Woojae Choi, Danil Kim

**Affiliations:** 1 Department of Farm Animal Medicine and Institute of Veterinary Science, College of Veterinary Medicine, Seoul National University, Seoul 08826, Republic of Korea; leegon1213@snu.ac.kr; 2 Farm Animal Clinical Training and Research Center, Institutes of Green-Bio Science and Technology, Seoul National University, Pyeongchang 25354, Republic of Korea; dura82@kangwon.ac.kr; 3 Department of Large Animal Medicine, College of Veterinary Medicine, Kangwon National University, Chuncheon 24341, Republic of Korea; 4 Cooperative Department of Veterinary Medicine, Faculty of Agriculture, Iwate University, Morioka 020-8550, Japan; akimura@iwate-u.ac.jp

**Keywords:** biosensor, three-axis accelerometer, bolus, reticular temperature, weaning period

## Abstract

**Simple Summary:**

Technological advancements and growing interest in farm animal health have led to the development of various biometric devices. Among these, bolus-type biosensors have been extensively used to monitor adult cattle, but their application in calves remains limited. In this study, we evaluated a newly developed miniature bolus biosensor, equipped with a triaxial accelerometer and temperature sensor, to monitor physiological changes in beef and dairy calves. The biosensor was orally administered shortly after birth and monitored for retention and data accuracy. Our findings showed that most calves have successfully retained the biosensor, which provided valuable data on calf activity, body temperature, and responses to the stressful weaning period. Significant changes in these parameters indicate the possibility of using such biosensors for early health monitoring. While occasional regurgitation of the device was noted, this study highlights the potential of these biosensors for improving calf health management, though further validation is needed.

**Abstract:**

In this study, a newly developed small wireless bolus biosensor, equipped with a triaxial accelerometer and temperature sensors, was applied to assess physiological changes in calves. The biosensor was orally implanted in calves, and its retention rate and location in the forestomach were examined. Data transmitted at 10 min intervals were analyzed to determine the characteristics of the calves at 10 and 100 days of age. Additionally, the daily averages of the vector magnitude (DV), changes in V over time (DV1), and reticular temperature (DRT) were analyzed during the experimental period. The biosensor was orally administered to twelve calves (six beef and six dairy) within 22 days of birth. Except for two regurgitated devices, the sensors transmitted data normally in a wireless manner for 15 weeks, recording physiological changes in the calves. The location of the biosensors was confirmed to be the reticulum. The analysis revealed that the V and V1 values were influenced by the physical characteristics of the biosensor’s location. During weaning, DV and DV1 values first increased and then decreased compared to pre-weaning, while the DRT increased post-weaning and remained elevated. These findings suggest that these types of biosensors can be used for monitoring calf health; however, further research is needed to determine their ability to detect pathological states.

## 1. Introduction

For a sustainable cattle industry, maximizing productivity through efficient management under limited conditions is crucial. Advances in dairy cow management technologies and their popularization have significantly increased the average volume of milk production per head globally over the past few decades [1,2]. Subsequently, advanced technologies and devices have been introduced to support this increase in milk production [3]. The robotic milking system is an ideal example of this progress, with a capability that extends beyond simply assisting the milking process to acquiring biometric information on each cow and using that information for feeding management [4]. Wearable information and communication technology (ICT) devices for obtaining biometric data are also widely used in the dairy industry [3], to detect the precise timing of estrus and parturition and to monitor for abnormal values such as body temperature, ruminoreticular motility, and rumen pH which are associated with reduced milk production [5]. Data are transmitted wirelessly, and through conversion, they communicate information about the physiological and pathological conditions of the animal to the owner, providing recommendations on health management and patient care [6].

However, in the beef cattle industry, the use of ICT devices is limited compared to that in the dairy cattle industry, focusing mainly on estrus detection [3,4]. On the other hand, one significant factor that reduces productivity in the beef cattle industry is calf mortality [7]. The mortality rate of calves under one year of age was reported to be an average of 5.7% on a large-scale beef farm over ten years in South Korea [8]. According to official data on Japanese Black cattle, the calf mortality rate until approximately the weaning period (during the first 180 days) is about 3.3%, with deaths primarily attributed to gastrointestinal and respiratory infectious diseases [9]. Calf mortality is also problematic in dairy calves, as revealed in a study involving a large dairy farm in South Korea in which an average mortality rate of 10.7% for calves under one year of age was recorded over a 10-year period [7]. Furthermore, the combined mortality–culling rate for female dairy calves and replacement heifers until their first calving was 21.2% in China, based on data collected from 31 farms located in various regions across the country [10]. These findings indicate that to achieve sustainability in the cattle industry, productivity can be improved by reducing calf mortality through early diagnosis and appropriate treatment. However, in Korea’s beef cattle breeding system, where calves live with their dams until weaning, the early detection of diseases, primarily through farmers’ observation is a challenging task due to practical difficulties such as limited time and insufficient labor in monitoring livestock effectively.

As calf diseases significantly impact productivity [7,9], continuous efforts are being made to improve early disease detection. As part of these efforts, sensors have recently been used to measure vital signs, and data analyses have facilitated the remote detection of pathophysiological states and the health condition of individuals or herds [11]. Since the main causes of mortality in calves are digestive and respiratory diseases before and after weaning [12], ICT devices should be used to provide data regarding features such as calf activity, digestive system motility, and body temperature in a wireless manner [11]. These metrics are crucial, as deviations from normal ranges can indicate potential health issues [13]. Consequently, information from these biometric data will allow farmers to identify diseased calves more rapidly than through observations. However, very few reports exist on the development and use of ICT devices specifically for calves.

In this study, we evaluated the feasibility of using a newly developed small bolus biosensor, equipped with three-axis acceleration and temperature sensors to monitor calf health. The sensor was applied to calves immediately after birth, and its retention rate was evaluated. We then analyzed the wirelessly transmitted sensor data and their converted values to determine the characteristics of each parameter. Finally, changes in data before and after weaning were analyzed.

## 2. Materials and Methods

### 2.1. Animals

The experiments were designed to evaluate whether physiological changes in healthy calves can be monitored by this new biosensor. Six Korean Native calves as beef calves (BCs) and six Holsteins calves as dairy calves (DCs) were selected and used sequentially in the order of their birth between September and December 2021. Although the primary objective of this study was to evaluate the biosensor’s performance in individual calves without a formal sample size calculation, we selected six calves from each group (beef and dairy calves) to enable the use of ANOVA and other statistical analyses.

The BCs were assessed at a breeding farm in Cheongju, a city in South Korea. They were reared together with dams in a 5 × 10 m barn with stanchions, with an extra 5 × 5 m space set aside to allow for the free movement of the calves. A concentrate-only diet was provided twice a day at 09:00 and 15:00 until one month after birth, and thereafter, hay and water were provided ad libitum. The amount of concentrate was gradually increased until each calf’s uptake reached 800 g/day, after which weaning was performed, i.e., when the calves were approximately 2–3 months old. For the prevention of calf diarrhea, the BC’s mothers were vaccinated twice, at 3 weeks and 6 weeks before parturition (ScourGuard^®^ 4k; Zoetis Animal Health, Parsippany, NJ, USA).

DCs were analyzed at a farm in Pyeongchang, Republic of Korea, and were reared individually in a calf hutch after birth. The calves were fed milk (2 L) twice daily (08:00 and 15:00) and the concentrate-only diet was provided ad libitum. After one month in the hutches, calves were reared together with other calves of the same age. Concentrate, hay, and water were provided ad libitum, and milk was provided twice daily until the calves reached three months of age. Then, the milk supply was stopped, and only concentrate and hay were provided.

### 2.2. Biosensor

**Figure 1:**
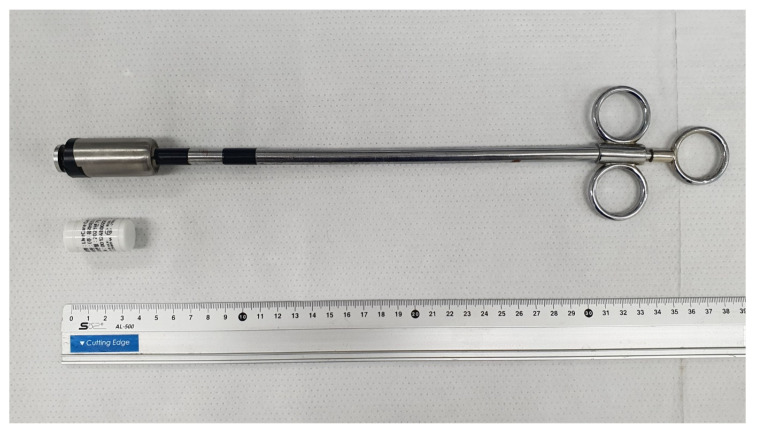
The biosensor is equipped with a triaxial accelerometer and a temperature sensor, along with its injector for oral administration in calves. A ruler is included for scale.

The biosensor used in this study (uLikeKorea Co. Inc., Seoul, Republic of Korea) was equipped with a triaxial accelerometer and a temperature sensor. It had a length of 35 mm, a diameter of 18 mm, a weight of 31 g, and a specific gravity (SG) of 3.48 (Figure 1). The biosensor’s center of gravity was located at its bottom. The longitudinal axis of the devices was considered to be the Z-axis, and the lateral and vertical directions of the long axis were used as the X and Y axes, respectively. The measuring range of the triaxial acceleration sensor was from −2 gravity to +2 gravity, and the measuring range of the temperature sensor was from −10 °C to +50 °C. Data from the biosensor were obtained at 10 min intervals and wirelessly transmitted every 30 min to the data receiver installed inside the farm using a low-power wide-area network. Only the highest vector (V) values for 10 min were transmitted and stored in the computer. The biosensor was inserted within three weeks after birth using a specially designed injector (Figure 1). Approximately 2, 3, 4, and 5 months after the biosensor was inserted, radiology tests were performed on seven calves (two DCs and five BCs) using a portable diagnostic X-ray unit (VET-20BT. POSKOM, Gyeonggi-do, Republic of Korea) to identify the location of the biosensor.

### 2.3. Acceleration Data Conversion

The root of the sum of squares of the accelerometer values of each axis (X-, Y- and Z) was measured as the following V value:

V = √(X^2^ + Y^2^ + Z^2^) (1)

The V value represents the magnitude of the acceleration applied to the triaxial acceleration sensor. If the sensor is in a fixed state without any additional force applied, the value represents gravitational acceleration. We converted the V value to V1 to represent the extent of change in the V value [14]. V1 is the root of the sum of squares of the differences between the measurement data of the current time (t) and the measured data of the previous time (t − 1) for each axis:

V1 = √{(X_t_ − X_t−1_)^2^ + (Y_t_ − Y_t−1_)^2^ + (Z_t_ − Z_t−1_)^2^} (2)

Since the values displayed on each axis of the accelerometer are greatly affected by gravity, the change in the position of the biosensor over time is well expressed by V1. To elucidate the characteristics of each parameter, raw and conversed data were analyzed at 10 (Day 10) and 100 days of age (Day 100) in each BC. Additionally, to confirm the daily change of data transmitted from each subject, the average daily data were obtained as the daily V (DV), daily V1 (DV1), and daily reticulum temperature (DRT).

### 2.4. Statistical Analysis

Changes in the daily data and weaning behavior are expressed as the mean ± standard deviation (SD). Since the normality test was not passed, a Wilcoxon signed-rank test was performed to compare data obtained for 24 h at 10 and 100 days of age. A repeated-measure one-way analysis of variance followed by Dunnett’s multiple-comparison test was performed to investigate any statistically significant differences between the pre-weaning (average for 5 days before weaning) and post-weaning time points for BCs. Since the highest V values for 10 min intervals were transmitted, data from most of the corresponding time points were used. In cases where missing data occurred, they were managed using listwise deletion, and normality was assessed using the Shapiro–Wilk test. All study results were analyzed using IBM SPSS Statistics for Windows, version 28 (IBM Corp., Armonk, NY, USA), and *p* < 0.05 was considered statistically significant.

## 3. Results

### 3.1. Retention and Location of the Biosensor

**Figure 2:**
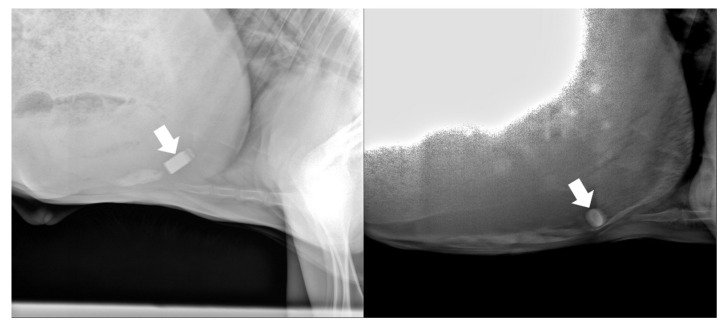
Representative images of lateral radiographs of the chest in 62-day-old (**left**) and 126-day-old (**right**) Korean Native beef calves showing the biosensor (white arrow). The images demonstrate that the biosensor was located in the reticulum and maintained its position throughout the experiment.

Out of the twelve animals, one BC and one DC regurgitated the biosensors, giving a biosensor retention rate of 83%. The biosensors were inserted into the BC and DC at 4 and 3 days of age and regurgitated at 59 and 32 days of age, respectively. The biosensor was re-inserted into the DC at 34 days of age and worked normally; however, re-insertion into the BC was unsuccessful due to a technical error. The remaining subjects (five BCs and five DCs) retained the biosensor, which functioned normally without any abnormalities. The results of radiological examination at 3 and 5 months old in DCs (*n* = 2) and at 2, 3, 4, and 5 months old in BCs (*n* = 5) confirmed that the biosensors were located in the reticulorumen (Figure 2).

### 3.2. Changes in the Biosensor Data throughout the Day

#### 3.2.1. Representative Triaxial Accelerometer Data in a Beef Calf on Days 10 and 100

**Figure 3:**
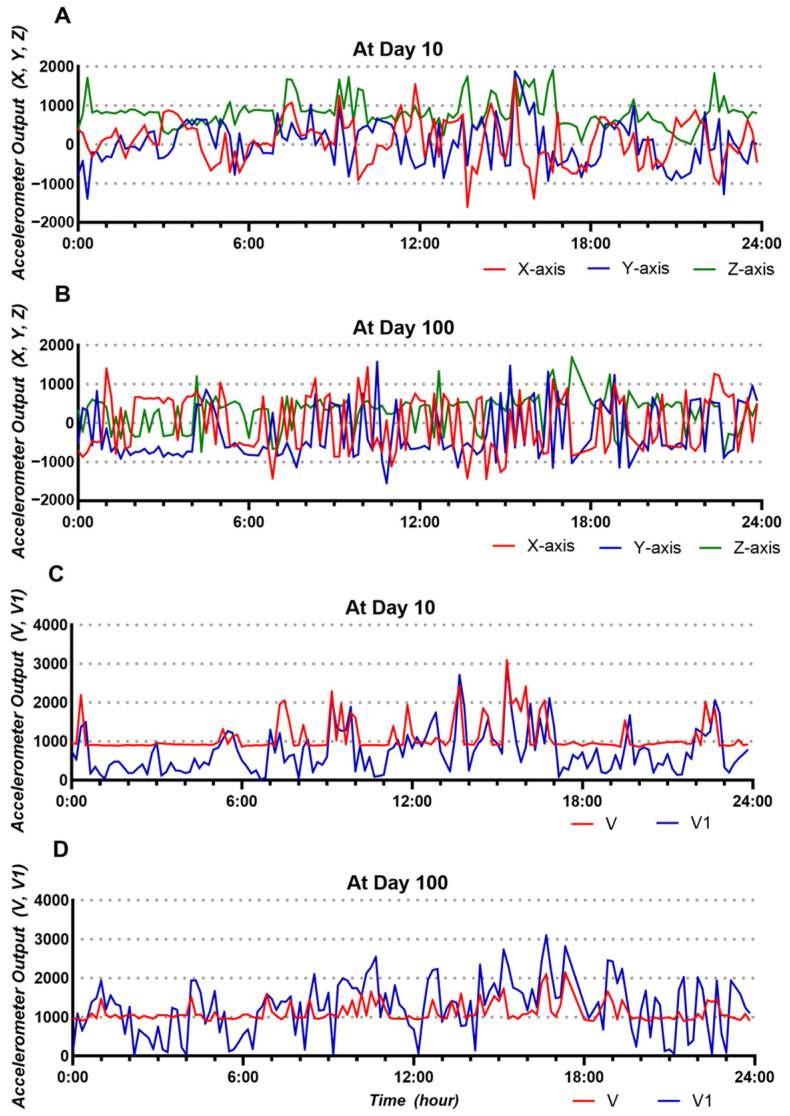
Representative raw triaxial acceleration data at Days 10 (**A**) and 100 (**B**) after birth, and the converted triaxial acceleration output at Days 10 (**C**) and 100 (**D**) from one beef calf (V: acceleration vector; V1: changes in V with time).

Representative raw data for each axis acquired from the triaxial accelerometer in one beef calf on Days 10 (A) and 100 (B) are shown in Figure 3. Both V and V1 calculated from the raw data are also presented (C, Day 10; D, Day 100). The raw data for the X, Y, and Z axes at Day 100 showed significantly greater fluctuations than those at Day 10 (Figure 3A,B). These fluctuation patterns were also observed in the changes in both V and V1 values at Day 100 (Figure 3C,D). The maximum V value on Day 10 was greater than that on Day 100, whereas the V1 value on Day 100 was greater than the maximum value. Between 01:00 and 05:00, a time when calves were least active, the V value was around 900 on Day 10, but it exceeded 1000 on Day 100.

#### 3.2.2. Frequency Distribution and Central Tendency Analysis of Accelerometer Data across Axes and Calculated Metrics on Days 10 and 100

**Figure 4:**
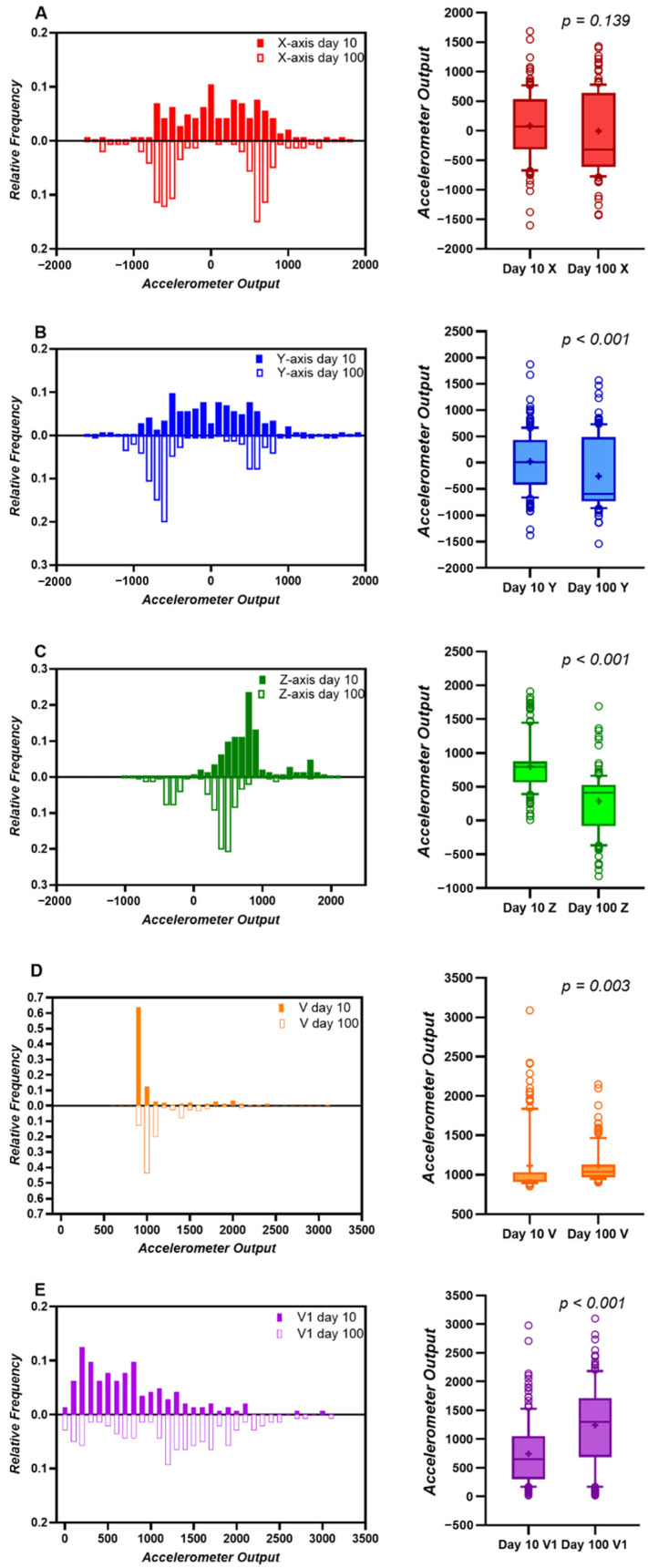
Relative frequency histogram (left) and box plot (right) showing the accelerometer output for each parameter (**A**): X-axis; (**B**): Y-axis; (**C**): Z-axis; (**D**): V; (**E**): V1 measured every 10 min for 24 h for one beef calf (No. 2) on Days 10 and 100 (bin width of the histogram: 100; V: acceleration vector, V1: changes in V over time).

The relative frequency histograms and box plots for each parameter (A, X-axis; B, Y- axis; C, Z-axis; D, V; E, V1) are presented in Figure 4. On Day 10, the frequency histogram of each axis is centered around 0, except for on the Z-axis, because of the biosensor’s center of gravity. On Day 100, the histogram almost disappears at around 0 and shows a bimodal pattern on all axes (Figure 4A–C, left). The median V and V1 values on Day 100 were significantly (*p* = 0.003 and *p* < 0.001, respectively) greater than those on Day 10 (Figure 4D,E, right).

#### 3.2.3. Developmental Changes in Dynamic Metrics and Stress Responses in Beef and Dairy Calves over Time

**Figure 5:**
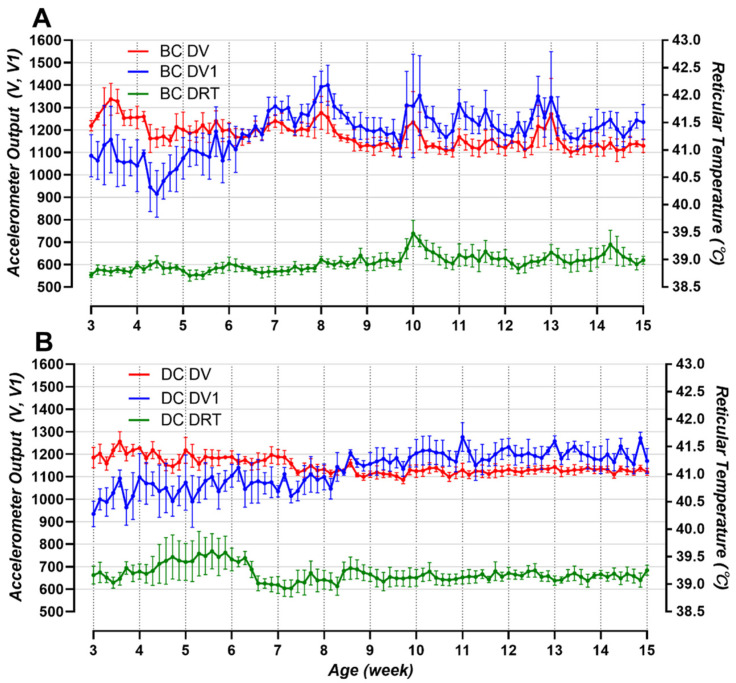
The daily mean DV, DV1, and DRT values for calves aged 3 to 14 weeks old were recorded daily for the BC (**A**) and DC (**B**) groups (mean ± SD, *n* = 5 for each **A** and **B**). The time from 0:00 to 23:50 was set as the daily standard (V: acceleration vector, V1: changes in V with time, BC: beef calf, DC: dairy calf, DV: daily V, DV1: daily V1, and DRT: daily reticulum temperature).

Figure 5 shows the changes in the average DV, DV1, and DRT for the five BCs (Figure 5A) and five DCs (Figure 5B) from 3 weeks to 14 weeks of age. In the DCs, the DRT was high at 4.5 to 6.5 weeks of age, when the animals were taken from individual hutches and reared together. In contrast, in the BCs, which were weaned during a similar period, an increase in body temperature was observed at around 10 weeks. The fluctuations in DV occurred within a certain range, but the DV1 continued to increase until about 8 weeks of age in both DCs and BCs and then fluctuated within a certain range.

### 3.3. Changes in the Biosensor Data in the Peri-Weaning Period

**Figure 6:**
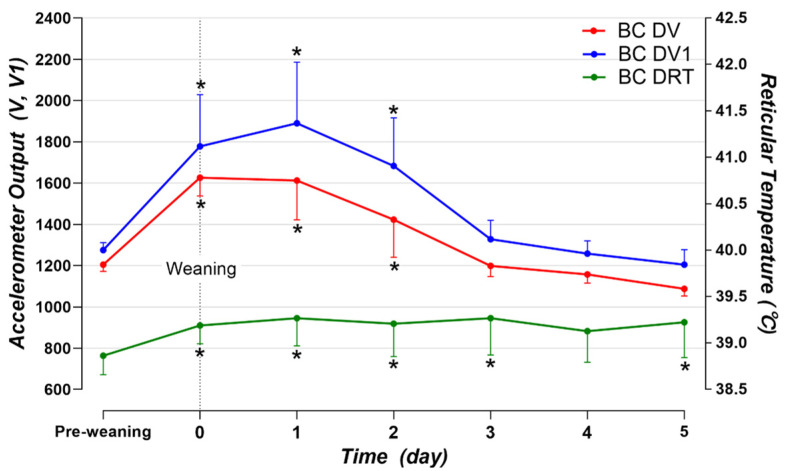
The average DV, DV1, and DRT values for five BCs during the five days before and five days after weaning (−5d to +5d). The values represent the mean ± SD. Significant differences between the pre-weaning values (average from −5d to −1d) and the values for each day after weaning are indicated as * *p* < 0.05 (V: acceleration vector, V1: changes in V over time, BC: beef calf, DV: daily V, DV1: daily V1, and DRT: daily reticulum temperature).

This experiment was performed only in BCs because it was difficult to specify the timing of weaning in DCs due to rearing management. To confirm the changes in data according to the weaning status, we considered the average value for five days before weaning as pre-weaning and examined the changes in data after weaning for six days. Statistically significant changes were observed for the DV (*p* < 0.001), DV1 (*p* < 0.001), and DRT (*p* = 0.03) compared with the respective pre-weaning values. The mean DV, DV1, and DRT values significantly increased immediately after weaning compared to pre-weaning, and this trend lasted for 3 days (Figure 6). Thereafter, DV and DV1 levels returned to pre-weaning levels, but DRT values remained significantly higher.

## 4. Discussion

The early detection of health problems is essential for disease treatment and control in the livestock industry [13,15,16]. Various ICT devices, such as ear-mounted, neck-collar, and bolus sensors, have been developed for disease detection [3,4]. Some commercially available bolus biosensors with temperature sensors and accelerometers have also been used, albeit only in adult cows. Previous studies confirmed the possibility of carrying out bovine respiratory disease detection by inserting a bolus body temperature sensor in calves, but their results were limited to measuring changes in body temperature [17,18]. Thus, the purpose of this study was to evaluate the feasibility of using a bolus sensor equipped with a temperature sensor and an accelerometer to assess calf health. After implanting the biosensor in calves, we successfully monitored its retention and analyzed the transmitted data to interpret physiological changes and evaluate overall health status.

In this study, out of the twelve calves in which the biosensor was implanted, 16.7% of calves regurgitated the biosensor. In comparison, a study related to bolus devices for electronic identification showed a retention rate of 99.7% for boluses manufactured with ceramic materials with an SG of over 3.3 [19]. The results of other studies suggest that a bolus with a minimum SG of 3 or higher is preferred to prevent regurgitation [20,21]. However, according to one study that confirmed the retention rate, an SG of 3 or higher does not guarantee permanent retention [22]. Using the retention rate equation derived from a previous study [22], a retention probability of 90% was observed in this study. Considering the limited number of subjects in this study and the difference in breed, further validation is required to account for other factors that affect the retention rate prediction in a larger and more diverse population.

The biosensor was located at the presumed reticulum in all subjects. In contrast, in a previous study, 91% (970/1068) of boluses were retrieved from the reticulum when a cylindrical bolus (external diameter, 20 mm; length, 66 mm; weight, 65 g) was inserted in calves under six weeks of age [19]. In another study, more than 90% of boluses (external diameter, 15 mm; length, 105 mm; density of 2.45 and 2.75) were found inside the reticula of slaughtered subjects [21]. However, Antonini et al. showed that when a bolus (external diameter, 17 mm; length, 67 mm; weight, 51.4 g) was inserted within three days to three weeks, one was lost, three out of seven were in the reticulum, and four were in the rumen [23]. Considering the possibility of the presence of the biosensor at a location other than the reticulum, additional research is needed to determine the retention rate of the biosensor in the reticulum and the changes in biosensor data at another site.

The raw data for each axis on Day 10 fluctuated less than those on Day 100 (Figure 3A,B), and a monomodal pattern was observed in the relative frequency histogram (Figure 4A–C). Because the triaxial accelerometer is greatly affected by gravity, changes in the posture of the biosensor due to rotation on the longitudinal or transversal axes significantly affect the acceleration value of each axis. Thus, the monomodal pattern seen on Day 10 may mean that the biosensor does not have enough space to ensure mobility or provide sufficient ruminoreticular motility. On the other hand, both median V and V1 values on Day 100 were significantly higher than those measured on Day 10. These results indicate that the force applied to the biosensor on Day 10 was greatly influenced by calf movement since, on Day 10, the reticulum did not have enough space to allow for the movement or rotation of the biosensor. The size of the calf reticulum on Day 100 was sufficient, so the biosensor moved or rotated due to the forces applied to the biosensor, resulting from factors such as calf movement and ruminoreticular contraction. However, the maximum force directly applied to the biosensor was less than that on Day 10, probably due to the fluid in the reticulum cushioning the movement of the biosensor. This may also be the reason for the stable V value within a certain range after this time point.

Ensuring sufficient space for biosensor movement is essential. The results shown in Figure 5 illustrate the changes in DV, DV1, and DRT. The DV exhibited constant fluctuations within a certain range in both BCs and DCs without significant changes, whereas the DV1 increased over the first eight weeks. Thereafter, the DV1 also exhibited constant fluctuations in both cow types within a specific range of approximately 1100 to 1300 in terms of acceleration output. At birth, the reticulorumen, omasum, and abomasum account for 38, 13, and 49% of a cow’s weight, respectively, and after eight weeks, these proportions reach 61%, 13%, and 25%. Subsequently, at 12 to 16 weeks, these values are 67, 18, and 18%, respectively [24]. Although the volume of the reticulorumen itself continues to expand as it grows, the DV1 value shown in Figure 5A,B remains at a constant level, which provides evidence that there is sufficient space for biosensor movement at eight weeks in BCs and eleven weeks in DCs. The increase in V1 values in both BC and DC due to the increase in reticulum volume shows a trend similar to the increase in rumen fill and sound, an indicator used in physical examinations to evaluate rumen development [25].

In South Korea, weaning usually occurs at approximately three months of age in beef calves, and it is a stressful period for animals as it is carried out before the natural weaning age, with an apparent distress response persisting for several days [26,27,28]. In previous research, after abrupt separation from the mother, calves vocalized at 41.9 calls/h for three days post-weaning, and their step count increased more than three times immediately after weaning compared to the day before weaning before returning to pre-weaning values on day three [26]. Similarly, in this study, the DV and DV1 values returned to their pre-weaning levels on day three after weaning. It is difficult to accurately determine whether the high DV and DV1 after weaning reflect the movement of internal organs due to vocalization or the increased external activity after weaning. Further research is necessary to understand the biosensor data derived from external activity. However, it was confirmed that DV and DV1 are indicative of the stressful and rapidly changing state in the weaning process. Additionally, the daily intake of milk or milk replacer is another key factor influencing the development of the digestive tract in lactating calves. In this study, calves were fed 2 L of milk twice daily, with ad libitum access to concentrates. However, the debate over the optimal amount of milk for calf consumption remains unresolved [29]. Future studies should evaluate the effects of different milk intake levels on ruminal development to better understand the relationship between milk consumption and digestive tract maturation.

Weaning was confirmed not only with the data from the triaxial accelerometer but also with the reticular temperature change. There was an increase in DRT immediately after weaning compared to pre-weaning, and this value remained significantly elevated for a few days. Similarly, in other studies, rectal temperature measurements revealed that the body temperature increased after weaning [27,30]. However, this measurement method has limitations due to difficulties in the sampling process compared to temperature measurement using a biosensor inserted in the forestomach [31,32]. Although rectal and rumen temperatures differ, various studies have confirmed a moderate-to-strong association between the two [32,33,34,35]. To the current authors’ knowledge, no study has investigated the precise changes in a calf’s reticular temperature during abrupt weaning when measured continuously every 10 min. The rapid increase in DRT observed after weaning is believed to be mainly caused by changes in behavior and stress immediately after weaning [26,36,37]. Additionally, it has been confirmed that the concentrations of stress hormones and carbohydrate metabolites increase after weaning [27,30,38]. Changes in behavior and stress can increase the heart rate and body temperature, and an increase in body temperature due to stress has been confirmed in several other animals [38,39,40]. Another possible reason for the alterations in the DRT may be due to changes in feeding immediately after weaning [41]. The forestomach temperature is correlated with the heat of fermentation in the rumen and is achieved through complex interactions involving changes in the core temperature [42]. In calves reared separately from birth, a rapid increase in the intake of concentrate feed was confirmed immediately after weaning [41]. Thus, the primary reasons for the increase in DRT after weaning may be stress or behavior changes due to weaning. This is beneficial for understanding the changes in the health or physiological conditions of the calves.

In terms of study limitations, there is a limit to the biosensor volume that can be applied to young individuals to reduce the loss of the biosensor and prevent the need for re-insertion, and it is difficult to control the SG of the biosensor comprising the triaxial accelerometer, body temperature sensor, and battery within this restricted volume. These constraints can impact the retention and performance of the biosensor. Therefore, further studies are needed to explore how to effectively measure physical activity using sensors under these limited conditions and how data from biosensors can be used to infer pathological conditions in calves. Addressing these limitations and considering other factors affecting biosensor retention and data accuracy in a larger and more diverse population will be crucial to fully realizing the potential of this technology in calf health monitoring.

## 5. Conclusions

This study evaluated the feasibility of the application of a newly developed calf biosensor equipped with three-axis acceleration and temperature sensors. The biosensor exhibited an acceptable retention rate in the reticulum. The data transmitted from the sensor provided useful information for interpreting physiological changes in healthy calves, and changes before and after weaning were also significant. These findings suggest that biosensors can provide valuable insights into calf health. Further studies are needed to determine whether data from biosensors are beneficial for inferring pathological conditions in calves.

## Data Availability

The original contributions presented in the study are included in the article. Further inquiries can be directed to the corresponding authors.

## References

[1] Schuster J.C., Barkema H.W., De Vries A., Kelton D.F., Orsel K. (2020). Invited review: Academic and applied approach to evaluating longevity in dairy cows. J. Dairy Sci..

[2] VandeHaar M.J., St-Pierre N. (2006). Major Advances in Nutrition: Relevance to the Sustainability of the Dairy Industry. J. Dairy Sci..

[3] Lee M., Seo S. (2021). Wearable Wireless Biosensor Technology for Monitoring Cattle: A Review. Animals.

[4] Stygar A.H., Gómez Y., Berteselli G.V., Dalla Costa E., Canali E., Niemi J.K., Llonch P., Pastell M. (2021). A Systematic Review on Commercially Available and Validated Sensor Technologies for Welfare Assessment of Dairy Cattle. Front. Vet. Sci..

[5] Nogami H., Arai S., Okada H., Zhan L., Itoh T. (2017). Minimized Bolus-Type Wireless Sensor Node with a Built-In Three-Axis Acceleration Meter for Monitoring a Cow’s Rumen Conditions. Sensors.

[6] Arai S., Okada H., Sawada H., Takahashi Y., Kimura K., Itoh T. (2019). Evaluation of ruminal motility in cattle by a bolus-type wireless sensor. J. Vet. Med. Sci..

[7] Hur T.-Y., Jung Y.-H., Choe C.-Y., Cho Y.-I., Kang S.-J., Lee H.-J., Ki K.-S., Baek K.-S., Suh G.-H. (2013). The dairy calf mortality: The causes of calf death during ten years at a large dairy farm in Korea. Korean J. Vet. Res..

[8] Kim U.-H., Jung Y.-H., Choe C., Kang S.-J., Chang S.-S., Cho S.-R., Yang B.-C., Hur T.-Y. (2015). Korean native calf mortality: The causes of calf death in a large breeding farm over a 10-year period. Korean J. Vet. Res..

[9] Sasaki S., Miki Y., Ibi T., Wakaguri H., Yoshida Y., Sugimoto Y., Suzuki Y. (2021). A 44-kb deleted-type copy number variation is associated with decreasing complement component activity and calf mortality in Japanese Black cattle. BMC Genom..

[10] Zhang H., Wang Y., Chang Y., Luo H., Brito L.F., Dong Y., Shi R., Wang Y., Dong G., Liu L. (2019). Mortality-Culling Rates of Dairy Calves and Replacement Heifers and Its Risk Factors in Holstein Cattle. Animals.

[11] Lowe G.L., Sutherland M.A., Waas J.R., Schaefer A.L., Cox N.R., Stewart M. (2019). Physiological and behavioral responses as indicators for early disease detection in dairy calves. J. Dairy Sci..

[12] Lorenz I., Mee J.F., Earley B., More S.J. (2011). Calf health from birth to weaning. I. General aspects of disease prevention. Ir. Vet. J..

[13] Silva F.G., Conceição C., Pereira A.M.F., Cerqueira J.L., Silva S.R. (2023). Literature Review on Technological Applications to Monitor and Evaluate Calves’ Health and Welfare. Animals.

[14] Choi W., Ro Y., Hong L., Ahn S., Kim H., Choi C., Kim H., Kim D. (2020). Evaluation of ruminal motility using an indwelling 3-axis accelerometer in the reticulum in cattle. J. Vet. Med. Sci..

[15] Bowen J.M., Haskell M.J., Miller G.A., Mason C.S., Bell D.J., Duthie C.A. (2021). Early prediction of respiratory disease in preweaning dairy calves using feeding and activity behaviors. J. Dairy Sci..

[16] Sutherland M.A., Lowe G.L., Huddart F.J., Waas J.R., Stewart M. (2018). Measurement of dairy calf behavior prior to onset of clinical disease and in response to disbudding using automated calf feeders and accelerometers. J. Dairy Sci..

[17] Timsit E., Assié S., Quiniou R., Seegers H., Bareille N. (2011). Early detection of bovine respiratory disease in young bulls using reticulo-rumen temperature boluses. Vet. J..

[18] Voss B., Laue H.J., Hoedemaker M., Wiedemann S. (2016). Field-trial evaluation of an automatic temperature measurement device placed in the reticulo-rumen of pre-weaned male calves. Livest. Sci..

[19] Caja G., Conill C., Nehring R., Ribó O. (1999). Development of a ceramic bolus for the permanent electronic identification of sheep, goat and cattle. Comput. Electron. Agric..

[20] Fallon R.J. (2001). The development and use of electronic ruminal boluses as a vehicle for bovine identification. Rev. Sci. Tech..

[21] Fallon R.J., Rogers P.A.M. (2001). Evaluation of Rumen Boluses as a Method of Electronic Animal Identification. Ir. J. Agric. Food Res..

[22] Ghirardi J.J., Caja G., Garín D., Casellas J., Hernández-Jover M. (2006). Evaluation of the retention of electronic identification boluses in the forestomachs of cattle. J. Anim. Sci..

[23] Antonini C., Trabalza-Marinucci M., Franceschini R., Mughetti L., Acuti G., Faba A., Asdrubali G., Boiti C. (2006). In vivo mechanical and in vitro electromagnetic side-effects of a ruminal transponder in cattle. J. Anim. Sci..

[24] Diao Q., Zhang R., Fu T. (2019). Review of Strategies to Promote Rumen Development in Calves. Animals.

[25] Schwarzkopf S., Kinoshita A., Hüther L., Salm L., Kehraus S., Südekum K.-H., Huber K., Dänicke S., Frahm J. (2022). Weaning age influences indicators of rumen function and development in female Holstein calves. BMC Vet. Res..

[26] Haley D.B., Bailey D.W., Stookey J.M. (2005). The effects of weaning beef calves in two stages on their behavior and growth rate. J. Anim. Sci..

[27] Lynch E.M., Earley B., McGee M., Doyle S. (2010). Effect of abrupt weaning at housing on leukocyte distribution, functional activity of neutrophils, and acute phase protein response of beef calves. BMC Vet. Res..

[28] Weary D.M., Jasper J., Hötzel M.J. (2008). Understanding weaning distress. Appl. Anim. Behav. Sci..

[29] Morrison S.J., Wicks H.C., Fallon R.J., Twigge J., Dawson L.E., Wylie A.R., Carson A.F. (2009). Effects of feeding level and protein content of milk replacer on the performance of dairy herd replacements. Animal.

[30] O’Loughlin A., McGee M., Doyle S., Earley B. (2014). Biomarker responses to weaning stress in beef calves. Res. Vet. Sci..

[31] Buckham Sporer K.R., Weber P.S., Burton J.L., Earley B., Crowe M.A. (2008). Transportation of young beef bulls alters circulating physiological parameters that may be effective biomarkers of stress. J. Anim. Sci..

[32] Rutherford N.H., Gordon A.W., Lively F.O., Arnott G. (2019). The Effect of Behaviour and Diet on the Rumen Temperature of Holstein Bulls. Animals.

[33] Lees A.M., Lees J.C., Lisle A.T., Sullivan M.L., Gaughan J.B. (2018). Effect of heat stress on rumen temperature of three breeds of cattle. Int. J. Biometeorol..

[34] Lees A.M., Sejian V., Lees J.C., Sullivan M.L., Lisle A.T., Gaughan J.B. (2019). Evaluating rumen temperature as an estimate of core body temperature in Angus feedlot cattle during summer. Int. J. Biometeorol..

[35] Rose-Dye T.K., Burciaga-Robles L.O., Krehbiel C.R., Step D.L., Fulton R.W., Confer A.W., Richards C.J. (2011). Rumen temperature change monitored with remote rumen temperature boluses after challenges with bovine viral diarrhea virus and *Mannheimia haemolytica*. J. Anim. Sci..

[36] Enríquez D., Hötzel M.J., Ungerfeld R. (2011). Minimising the stress of weaning of beef calves: A review. Acta Vet. Scand..

[37] Price E.O., Harris J.E., Borgward R.E., Sween M.L., Connor J.M. (2003). Fenceline contact of beef calves with their dams at weaning reduces the negative effects of separation on behavior and growth rate. J. Anim. Sci..

[38] Loberg J.M., Hernandez C.E., Thierfelder T., Jensen M.B., Berg C., Lidfors L. (2008). Weaning and separation in two steps—A way to decrease stress in dairy calves suckled by foster cows. Appl. Anim. Behav. Sci..

[39] Adriaan Bouwknecht J., Olivier B., Paylor R.E. (2007). The stress-induced hyperthermia paradigm as a physiological animal model for anxiety: A review of pharmacological and genetic studies in the mouse. Neurosci. Biobehav. Rev..

[40] Vinkers C.H., Penning R., Hellhammer J., Verster J.C., Klaessens J.H., Olivier B., Kalkman C.J. (2013). The effect of stress on core and peripheral body temperature in humans. Stress.

[41] Scoley G., Gordon A., Morrison S. (2019). Performance and Behavioural Responses of Group Housed Dairy Calves to Two Different Weaning Methods. Animals.

[42] Beatty D.T., Barnes A., Taylor E., Maloney S.K. (2008). Do changes in feed intake or ambient temperature cause changes in cattle rumen temperature relative to core temperature?. J. Therm. Biol..

